# Tillage and nitrogen fertilization enhanced belowground carbon allocation and plant nitrogen uptake in a semi-arid canola crop–soil system

**DOI:** 10.1038/s41598-017-11190-4

**Published:** 2017-09-06

**Authors:** Jharna Rani Sarker, Bhupinder Pal Singh, Xinhua He, Yunying Fang, Guangdi D. Li, Damian Collins, Annette L. Cowie

**Affiliations:** 1 University of New England, Armidale, NSW 2351 Australia; 2 NSW Department of Primary Industries, Elizabeth Macarthur Agricultural Institute, Woodbridge Road, Menangle, NSW 2568 Australia; 3 College of Resources and Environment, Southwest University, Chongqing, 400715 China; 4 NSW Department of Primary Industries, Wagga Wagga Agricultural Institute, Wagga Wagga, NSW 2650 Australia; 5 NSW Department of Primary Industries, Beef Industry Centre, Trevenna Road, Armidale, NSW 2351 Australia

## Abstract

Carbon (C) and nitrogen (N) allocation and assimilation are coupled processes, likely influencing C accumulation, N use efficiency and plant productivity in agro-ecosystems. However, dynamics and responses of these processes to management practices in semi-arid agro-ecosystems are poorly understood. A field-based ^13^CO_2_ and urea-^15^N pulse labelling experiment was conducted to track how C and N allocation and assimilation during canola growth from flowering to maturity were affected by short-term (2-year) tillage (T) and no-till (NT) with or without 100 kg urea-N ha^−1^ (T-0, T-100, NT-0, NT-100) on a Luvisol in an Australian semi-arid region. The T-100 caused greater (P < 0.05) belowground C allocation and higher (P < 0.05) translocation of soil N to shoots and seeds, compared to other treatments. Microbial N uptake was rapid and greatest in the fertilized (*cf*. non-fertilized) treatments, followed by a rapid release of microbial immobilized N, thus increasing N availability for plant uptake. In contrast, management practices had insignificant impact on soil C and N stocks, aggregate stability, microbial biomass, and ^13^C retention in aggregate-size fractions. In conclusion, tillage and N fertilization increased belowground C allocation and crop N uptake and yield, possibly *via* enhancing root–microbial interactions, with minimal impact on soil properties.

## Introduction

Plants allocate recently photo-assimilated carbon (C) to aboveground and belowground organs to support their structural and non-structural components and metabolic processes^1, 2^, influencing the C source sink balance^3^ and nutrient cycling in terrestrial ecosystems^4^. Of the total belowground C allocation, a significant amount can be translocated from roots to soil (*e*.*g*. as exudates) and subsequently respired as CO_2_^5–7^. Root exudates are energy-rich substrates which influence the growth and activity of microbes, mineralisation of soil organic matter (SOM), and uptake of soil-released nitrogen (N) by plants^4^. Thus, belowground C allocation is a key process in influencing the coupled source–sink activities between shoots, roots and soil microorganisms with implications for C and N cycling, N use efficiency and biomass productivity in agro-ecosystems^2, 8, 9^. However, the responses of these agro-ecosystem dynamics to management practices, including the relationship between belowground C allocation and crop N uptake, are not well understood^4, 7, 10^. A better understanding is needed of the potential of management practices to enhance plant C input and N use efficiency in crop–soil systems^7^. Furthermore, research on the impact of agricultural management on soil C and N dynamics is critical for the sustainability of agro-ecosystems and the quality of the environment^11–14^.

There are studies that have examined allocation dynamics of newly assimilated C and N and their retention in aboveground and belowground pools under field and controlled conditions^7, 10, 15^. For example, in a recent field study, An *et al*.^10^ reported 12–15% allocation of the newly assimilated ^13^C to belowground pools (such as soil, roots and microbial biomass) 15 days after pulse labelling in differently managed maize–soil systems. Ge *et al*.^15^ reported 8–19% allocation of newly assimilated ^14^C to belowground pools 36 days after continuous labelling in differently managed rice–soil systems under controlled conditions. Further, in another recent field study under a dryland condition, Fang *et al*.^7^ reported 1–2% allocation of the newly assimilated ^13^C to belowground pools and 0.5–0.7% translocation of the new soil-released ^15^N to aboveground pools 50 days after pulse labelling in differently managed wheat–soil systems. Yet, less attention has been given to the understanding of how different management practices influence whole-plant C and N allocation, assimilation and interactions under field conditions^7^, particularly in semi-arid dryland agro-ecosystems, where 50% or more of plant available N is derived from SOM mineralisation^16^. Furthermore, dryland regions impart severe constraints to crop productivity, due to low moisture availability, low soil C and climatic variability^7, 17, 18^. It is thus important to identify how management practices could enhance key ecosystem processes and functions, such as mutualistic relationships between shoots, roots and microorganisms in relation to belowground C allocation, plant N use efficiency, crop yield, and retention of belowground allocated C in soil aggregates. *In situ* monitoring techniques using dual stable isotopic (^13^C and ^15^N) pulse labelling can allow to quantify such ecosystem processes and functions in contrastingly managed crop–soil systems^4, 7, 19, 20^. Many studies have reported that a tracing period of more than one to several weeks after ^13^C pulse labelling is appropriate to achieve an equilibrium partitioning of the newly assimilated C in plant–soil pools^7, 10, 21–23^. Further, a recent study has shown that a tracing period of several weeks can be useful to achieve an equilibrium partitioning of new C and N in plant–soil pools, and thus to provide a realistic assessment of the impact of management practices on the coupling between C allocation and N use efficiency in crop–soil systems^7^.

It has been proposed that soil aggregates can stabilize root- and microbial-derived C and N through physical and chemical mechanisms and may increase their stocks in soil^24, 25^. Studies reported that the allocation, fate and retention of newly added C and N can vary among different aggregate-size classes^7, 26^. For example, Fang *et al*.^7^ reported that after 50 days of pulse labelling, the new C and N retention was higher in micro-structures relative to larger-sized aggregates, likely due to less accessibility of SOM to microbes. Additionally, the processes of C and N accumulation in soil aggregates may also be related to the extent of belowground C allocation, which may concurrently enhance microbial activity, N cycling and soil aggregate stability^7, 26, 27^. As these soil processes may be impacted by tillage and N fertilization^15, 28^, a better understanding of the allocation, fate and retention of C and N in soil aggregates of different size classes is needed to acquire insights into pathways of SOM accumulation under contrastingly managed cropping systems.

Canola is the Australia’s third-largest broadacre crop after wheat and barley^29^. Canola is grown as a key rotation crop across the wheat belt areas of different countries^29–31^. Canola generally has a high N requirement to maintain adequate seed yield and quality^32, 33^. Further, studies have reported that, at the reproductive stage, C allocation to belowground pools may decrease due to increasing demand for the plant-assimilated C by the reproductive pools such as flowers, pods and seeds^21, 34^. However, some of the assimilated C may still be allocated belowground at the critical (reproductive) growth stage to meet the plant’s nutrient and water demand^35, 36^. This study therefore aimed to assess the impact of tillage intensity and N fertilization on the coupling between aboveground and belowground pools in relation to assimilation and allocation of new C and N, in a canola crop–soil system. *In situ* plant ^13^C and soil ^15^N labelling was performed at flowering to examine the flow of newly assimilated C and N in the crop−soil system until grain maturity, and to quantify their distribution in plant pools, microbial biomass and aggregate-size fractions in a Chromic Luvisol.

No-till is a widely adopted practice in Australian dryland cropping systems, providing benefits such as improvement in soil structure, water retention and SOC^14, 37^. Conversely, no-till farming may cause soil compaction and increase weed infestation^38^. It is also being recognised that relatively low-intensity tillage operations (*e*.*g*. scarifier, harrowing, discing), as performed in our study, may create a soil environment favourable for germination, root proliferation, soil microbial activity and plant growth, with minimal impact on soil structure and SOC^39–41^. Additionally, N fertilization can support crop growth and yield^14^ and may influence belowground C allocation and soil microbial activity^42, 43^. Thus, we hypothesized that (i) tillage with N fertilization could increase belowground C allocation through enhancement of root growth, stimulating microbial activity and interactions, compared to tillage without N fertilization and no-till with and without N, leading to the greatest plant N uptake from soil; (ii) uptake of soil-released N by microbes could be more prominent under tillage and N fertilization than under no-till (with and without N) and tillage without N; and (iii) tillage (*vs*. no-till) would have minimal impact on aggregate stability and C and N storage in stable aggregates.

## Results

### Allocation of newly assimilated ^13^C and ^15^N in canola–soil system

**Figure 1:**
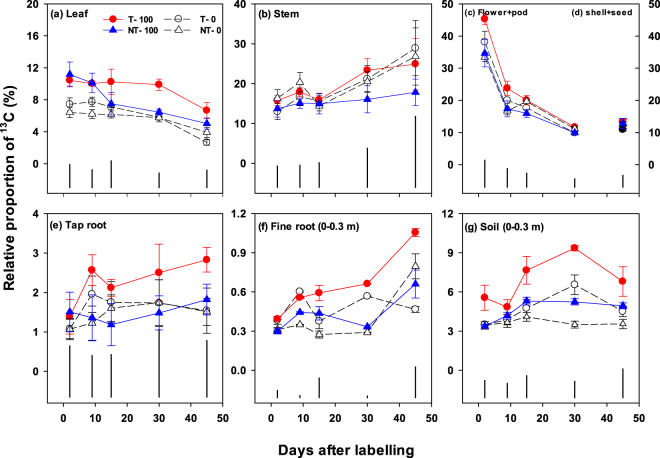
Relative proportion (%) of the pulse-added ^13^CO_2_-C recovered in the aboveground (**a**,**b**,**c**) and belowground pools (**d**,**e**,**f**)) in a canola crop–soil system from flowering to harvesting as affected by tillage (T) and no-till (NT) with or without 100 kg urea-N ha^−1^ (*i*.*e*. T–0, T–100, NT–0, NT–100). Error bars are ± standard errors (*n* = 3). Vertical black bars show least significant differences (at 5% level, LSD_0.05_) at different time points.

**Figure 2:**
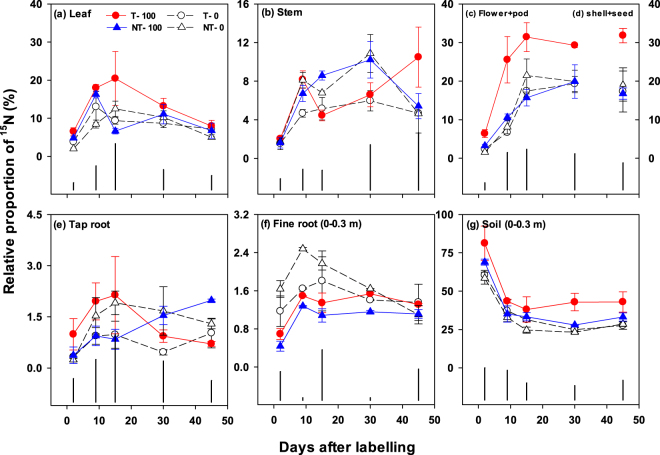
Relative proportion (%) of the pulse-added urea-^15^N recovered in the aboveground (**a**,**b**,**c**) and belowground pools (**d**,**e**,**f**)) in a canola crop–soil system from flowering to harvesting as affected by tillage (T) and no-till (NT) with or without 100 kg urea-N ha^−1^ (*i*.*e*. T–0, T–100, NT–0, NT–100). Error bars are ± standard errors (*n* = 3). Vertical black bars show least significant differences (at 5% level, LSD_0.05_) at different time points.

**Figure 3:**
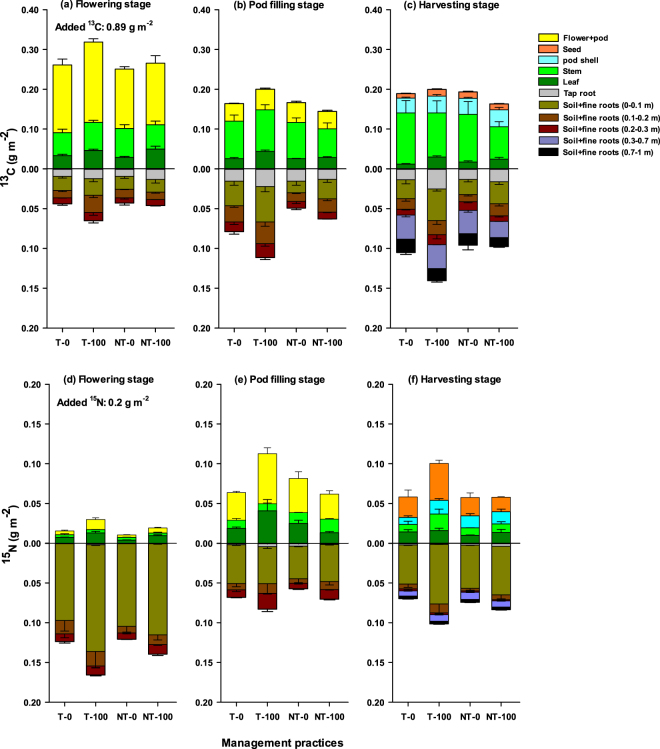
Partial budget (g m^−2^) of the pulse-applied ^13^C and^15^N allocation across the aboveground (*i*.*e*. leaf, stem, flower/seed) and belowground pools (*i*.*e*. tap root and soil plus fine roots to 1 m depth) under different management practices at flowering stage (day two) (**a**,**c**), pod filling stage (day thirty) (**b**,**d**), and harvesting stage (day forty five) (**c**,**f**). Error bars are ± standard errors (*n* = 3).

Pulse labelling with ^13^CO_2_ and ^15^N produced isotopically traceable assimilates that were allocated among aboveground and belowground pools immediately after pulse labelling (Figs 1, 2 and 3). Time had a significant impact on the allocation of newly assimilated C and N in all aboveground and belowground pools, except in the tap roots (Table 1).

**Table 1:** Results of repeated-measures ANOVA (P values) to test for overall effects of tillage, fertilizer, time and their interactions on ^13^C and ^15^N partitioning in crop–soil system.

	Tillage	Fertilizer	Time	Tillage × Fertilizer	Tillage × Time	Fertilizer × Time	Tillage × Fertilizer × Time
^**13**^ **C recovery in pools (%)**							
Leaf	0.264	<**0**.**001**	<**0**.**001**	0.097	0.270	0.567	**0**.**047**
Stem	0.921	0.742	**0**.**004**	0.161	0.785	0.555	0.508
Flower + pod	0.214	0.490	<**0**.**001**	0.068	0.097	0.361	0.608
Pod shell + seed (day 45)	0.551	0.233	—	0.302	—	—	—
Tap root	0.093	0.385	0.135	0.377	0.224	0.494	0.778
Fine roots (0–30 cm)	<**0**.**001**	<**0**.**001**	<**0**.**001**	**0**.**016**	<**0**.**001**	<**0**.**001**	<**0**.**001**
Soil (0–30 cm)	0.054	**0**.**001**	**0**.**001**	**0**.**043**	**0**.**001**	0.079	0.406
^**15**^ **N recovery in pools (%)**							
Leaf	**0**.**050**	**0**.**001**	**0**.**005**	0.937	0.693	0.342	0.206
Stem	**0**.**023**	0.132	<**0**.**001**	0.163	**0**.**008**	0.589	**0**.**036**
Flower + pod	**0**.**005**	<**0**.**001**	<**0**.**001**	**0**.**036**	0.416	0.168	0.075
Pod shell + seed(day 45)	**0**.**017**	**0**.**024**	—	**0**.**006**	—	—	—
Tap root	0.076	0.082	**0**.**042**	0.896	**0**.**041**	0.960	0.067
Fine roots (0–30 cm)	<**0**.**001**	<**0**.**001**	<**0**.**001**	<**0**.**001**	<**0**.**001**	<**0**.**001**	<**0**.**001**
Soil (0–30 cm)	**0**.**036**	**0**.**008**	<**0**.**001**	0.115	0.727	0.468	0.121
^**13**^ **C recovery in aggregates (%)**							
Mega-aggregates (>2 mm)	0.732	0.520	**0**.**021**	0.181	0.871	0.758	0.936
Macro-aggregates (0.25–2 mm)	**0**.**011**	**0**.**041**	<**0**.**001**	0.976	0.111	0.720	0.104
Micro-aggregates (<0.25 mm)	0.215	0.409	**0**.**003**	0.677	0.370	0.805	0.557
^**15**^ **N recovery in aggregates (%)**							
Mega-aggregates (>2 mm)	<**0**.**001**	**0**.**049**	<**0**.**001**	**0**.**01**	<**0**.**001**	**0**.**016**	<**0**.**001**
Macro-aggregates (2–0.25 mm)	**0**.**041**	<**0**.**001**	<**0**.**001**	**0**.**002**	**0**.**006**	**0**.**009**	0.128
Micro-aggregates (<0.25 mm)	**0**.**015**	**0**.**001**	<**0**.**001**	0.677	0.751	0.261	0.661
^**13**^ **C recovery in DOC (%)**	0.729	0.803	<**0**.**001**	0.492	0.766	0.797	0.957
^**13**^ **C recovery in MBC (%)**	0.052	0.122	<**0**.**001**	0.437	0.939	0.753	0.786
^**15**^ **N recovery in DN (%)**	**0**.**016**	<**0**.**001**	<**0**.**001**	0.131	0.272	<**0**.**001**	0.096

Values in bold highlight significant effects at P < 0.05. DOC = Dissolve organic carbon; DN = Dissolve nitrogen.

Two days after labelling, most of the added ^13^C remained in the aboveground pools, while a small proportion of the assimilated ^13^C was allocated belowground across all management practices (Fig. 1). Of the total added ^13^CO_2_ (0.89 g m^−2^), 6–11%, 14–16% and 33–45% were recovered within two days in the leaf, stem, and flower + pod pools, respectively, across the management practices (Figs 1a–c and 3a). The relative proportion of ^13^C decreased in the leaf (P < 0.001) and flower + pod pools (P < 0.001), while ^13^C allocation increased in the stem (P < 0.01) with time. At harvest, 2–6%, 18–29% and 11–13% were recovered in the leaf, stem, and shell + seed pools, respectively, across the management practices (Figs 1a–c and 3c). Over the chasing period, fertilization (P < 0.001) and its interaction with tillage and time had significant effects on new C allocation in the leaf (Table 1). Overall, the leaf ^13^C recovery was higher in the T–100 than the other treatments.

**Figure 4:**
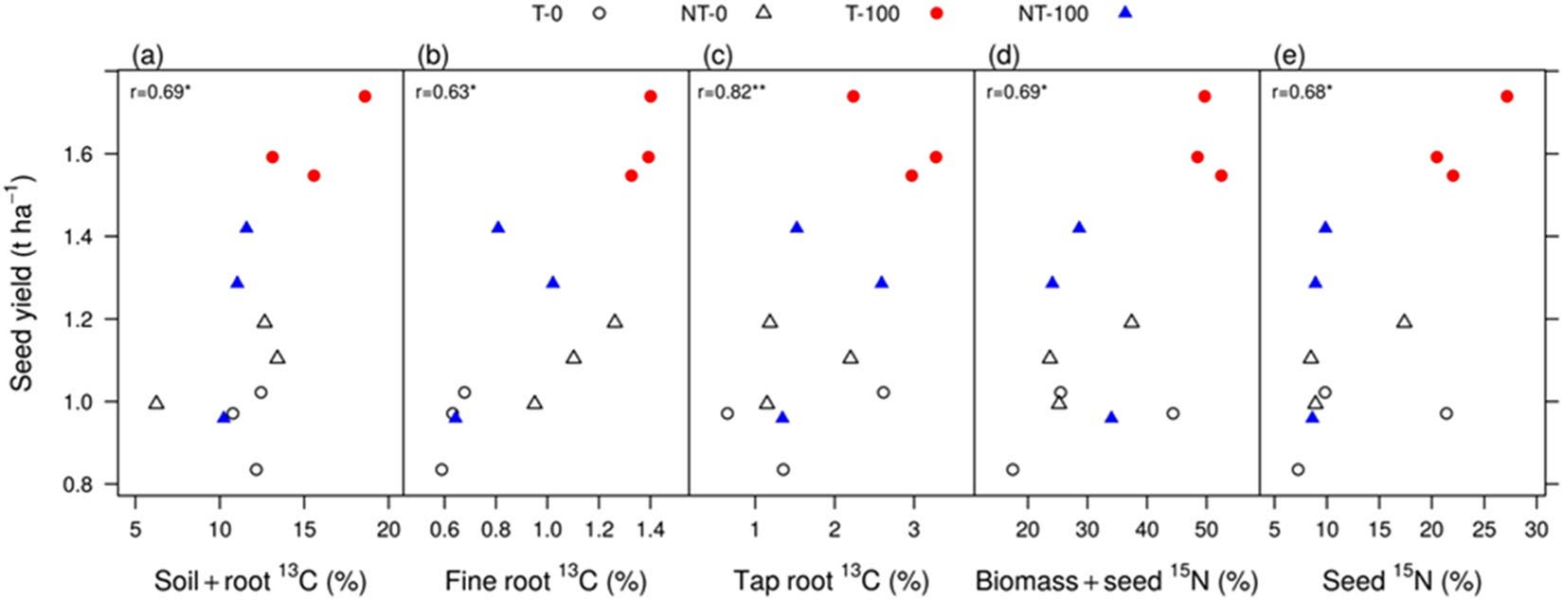
Relationships between (1) canola seed yield (t ha^−1^) and relative proportion (%) of belowground carbon (^13^C) allocation to soil plus roots to 0.1 m depth (**a**), fine roots to 0.1 m depth (**b**) and tap roots (**c**); and (2) canola seed yield and relative proportion of aboveground nitrogen (^15^N) translocation to biomass plus seed (**d**) and seed (**e**), as affected by tillage (T) and no-till (NT) with or without 100 kg urea-N ha^−1^ (*i*.*e*. T-0, T-100, NT-0, NT-100). *Significant correlation (P < 0.05); **highly significant correlation (P < 0.01).

Among the belowground pools 1.1–1.5%, 0.3–0.4% and 3.4–5.5% of the ^13^C was allocated to tap roots, fine roots (0–0.3 m depth) and soil (0–0.3 m depth), respectively, two days after labelling, across the management practices (Figs 1d–f and 3a). There were highly significant (P < 0.001) interactions between tillage, fertilizer and time in allocation to fine roots, but not in tap roots (Table 1). The ^13^C allocation to fine roots increased with time (P < 0.001), recovering 0.4–1.1% at harvest across the treatments (Fig. 1e). In the soil pool, fertilizer (P < 0.01), time (P < 0.01) and the interaction of fertilizer with tillage (P < 0.05), and time with tillage had significant (P < 0.01) effects on the ^13^C recovery (Table 1). After two days, the new C allocation in the soil either increased over the chasing period or stabilised among the management practices. At harvest, 3.0–7.0% of the added ^13^C was recovered in soil to 0.3 m depth. Overall, the new C allocation in fine roots and soil was highest in the T–100, relative to the other treatments. Further, at harvest, the aboveground biomass and seed yield was higher (P < 0.01) in the T–100 than the other treatments (Fig. S1). Although, there was a no clear relationship between the belowground C allocation and aboveground biomass, the belowground C allocation had a significant positive correlation with seed yield (Fig. 4).

**Figure 5:**
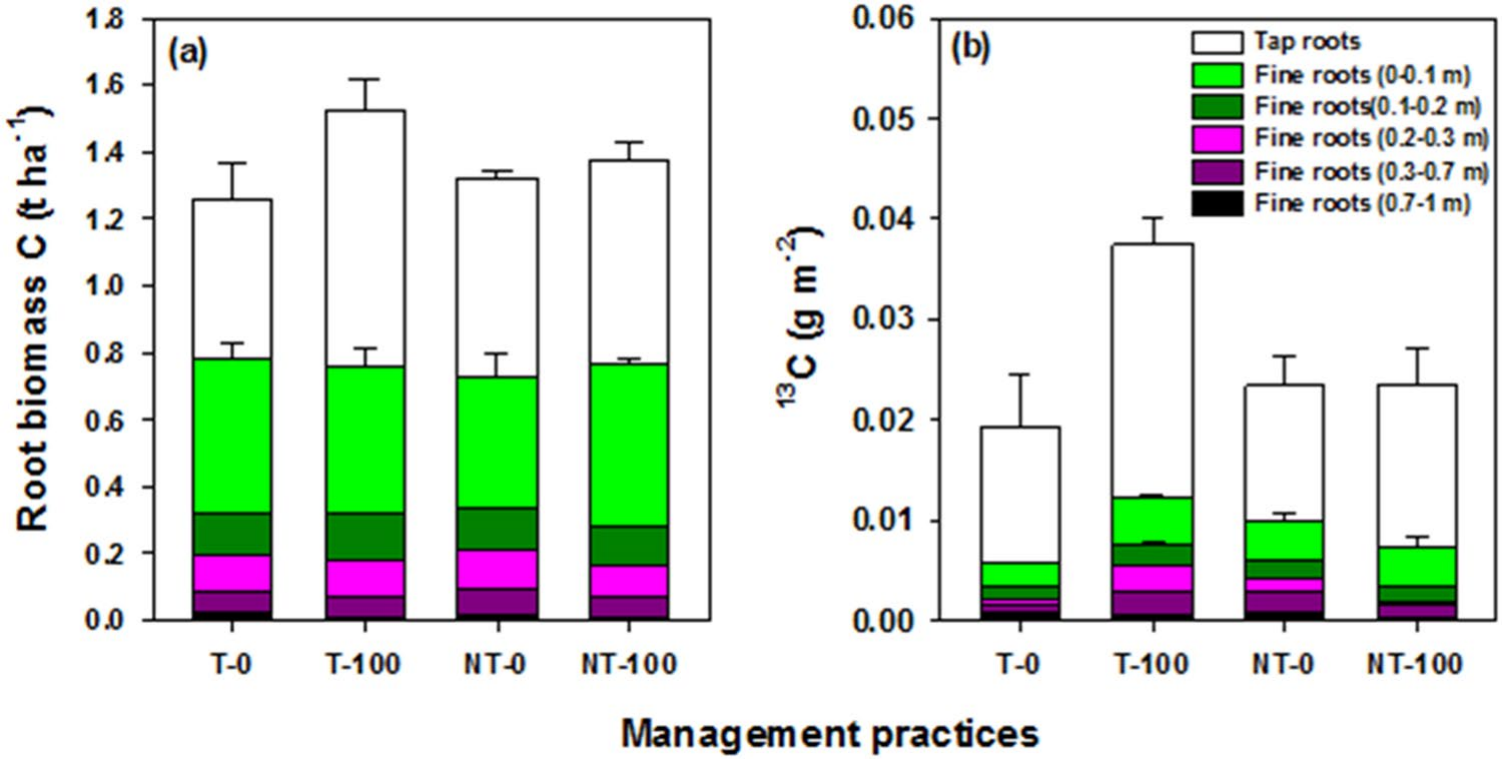
Root biomass (tap and fine roots) distribution (t ha^−1^) (**a**) and new ^13^C distribution (g m^−2^) in tap and fine roots (**b**), at 0–0.1 m, 0.1–0.2 m, 0.2–0.3 m, 0.3–0.7 m and 0.7–1 m soil depth at harvesting stage as affected by tillage (T) and no-till (NT) with or without 100 kg urea-N ha^−1^ (*i*.*e*. T-0, T-100, NT-0, NT-100). Error bars are ± standard errors (*n* = 3).

Of the total added amount (0.89 g ^13^C m^−2^), the net ^13^C recovery in the aboveground pools varied between 0.50 and 0.64 g m^−2^ (56–71%) across the management practices on day two. The T–100 showed higher ^13^C recovery than the other practices (Fig. 3a). The net ^13^C recovery in the aboveground pools then decreased rapidly over time, and at the pod filling stage (day 30), 0.30–0.40 g ^13^C m^−2^ (32–45%) was retained, which was similar to that recovered at harvest (0.32–0.40 g m^−2^) (Fig. 3b,c). The net ^13^C recovery in the belowground pools to 0.3 m depth varied between 0.03 and 0.05 g m^−2^ (4–6%) on day two, which increased to 0.04–0.09 g m^−2^ (4–10%) at pod filling, and remained similar (0.04–0.08 g m^−2^) at harvest across the treatments (Fig. 3). The results to 1.0 m depth at harvest showed additional ^13^C recovery of 0.002–0.003 g m^−2^ (0.2–0.3%) in the fine roots and 0.03–0.05 g m^−2^ (4–5%) in the soil + fine roots to 0.3–1.0 m depth across the treatments (Figs 3c and 5b).

The allocation of ^15^N in the aboveground and belowground pools showed the opposite pattern to that of ^13^C. On day two, the majority of soil-applied ^15^N (0.2 g ure﻿a-^15^N m^−2^) remained in the belowground pools, while a small proportion was translocated to the aboveground biomass in all management practices (Fig. 2). Of the total soil ^15^N, 2.0–6.5%, 1.5–2.0% and 1.5–6.0% was translocated to the leaf, stem, and flower + pod pools, respectively, within two days (Fig. 2a–c). However, with time, the allocation of soil ^15^N increased in the aboveground pools (Table 1; Fig. 3d–f). At harvest, 5–8%, 4–10% and 17–32% of the ^15^N was recovered in the leaf, stem, and shell + seed pools, respectively, across the treatments (Figs 2a–c and 3f). In the stem, ^15^N allocation was significantly impacted by the interactive effect of tillage × fertilizer × time (P < 0.05) (Table 1). In the leaf and flower + pod pools, tillage, fertilizer and time each had significant effects (P < 0.05) (Table 1). In contrast, ^15^N allocation in pod shell + seed was significantly impacted by the interacting effect of tillage × fertilizer (P < 0.05) (Table 1). The T–100 showed the highest allocation of ^15^N in flowers _+_ pods throughout (Fig. 2c) and there was a significant positive correlation between aboveground N translocation and seed yield (Fig. 4). Two days after labelling, 0.3–1.0% and 0.4–1.6% of the soil-applied ^15^N was translocated to tap and fine roots (0–0.3 m), respectively, and 58–81% was recovered in soil (0–0.3 m), which decreased over time (Figs 2d–f and 3d–f). Tillage, fertilizer, time and their interactions had a significant (P < 0.001) effect on the ^15^N recovery in fine roots, whereas, only tillage, fertilizer or time had a significant effect (P < 0.05) on the soil ^15^N recovery (Table 1). Overall, the T–100 resulted in higher soil ^15^N recovery than the other treatments. The ^15^N recovery in the soil decreased rapidly from the flowering to pod filling and then stabilised. At harvest, 27–43% of the ^15^N remained in the soil to 0.3 m depth (Fig. 2).

Across the practices, the net recovery of the added ^15^N (0.2 g urea-﻿^15^N m^−2^) in the aboveground pools varied between 0.01 and 0.03 g m^−2^ (5–15%) (Fig. 3d) on day two, and increased to 0.07–0.10 g m^−2^ (34–49%) at pod filling (Fig. 3e) and remained similar (0.06–0.10 g m^−2^) at harvest (Fig. 3f). On day two, the net recovery of ^15^N in the belowground pools to 0.3 m depth was 0.12–0.16 g m^−2^ (60–82%) (Fig. 3d). This recovery decreased to 0.06–0.09 g m^−2^ (29–44%) at harvest, while only 5–6% of the added ^15^N was recovered in the 0.3–1.0 m depth (Fig. 3f). The T–100 (*cf*. the other treatments) showed the highest ^15^N recovery in both the aboveground and belowground pools (Fig. 3d–f).

### ^13^C and ^15^N recovery in microbial biomass

**Figure 6:**
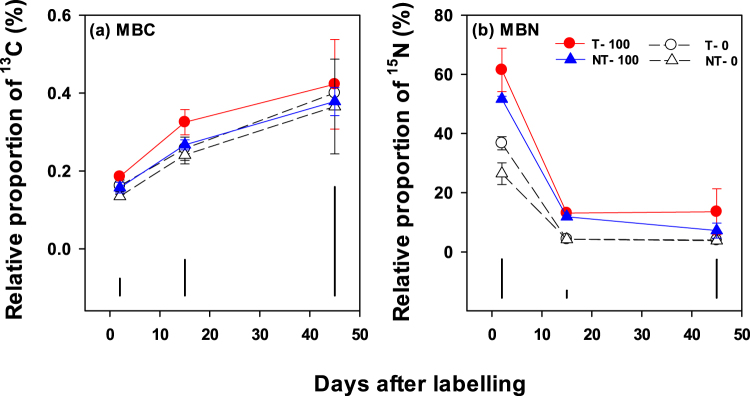
Relative proportion (%) of the pulse-added ^13^CO_2_-C in microbial biomass carbon (MBC) (**a**) and urea-^15^N in microbial biomass nitrogen (MBN) (**b**) in the 0–0.1 m soil from canola flowering to harvesting stage as affected by tillage (T) and no-till (NT) with or without 100 kg urea-N ha^−1^ (*i*.*e*. T-0, T-100, NT-0, NT-100). Error bars are ± standard errors (*n* = 3). Vertical black bars show least significant differences (at 5% level, LSD_0.05_) at different time points.

Tillage and fertilizer, and their interactions with time had no significant (P > 0.05) effects on the recovery of ^13^C in MBC and DOC (Table 1; Fig. S2a). At two days, 0.13–0.18% of the added ^13^C was recovered in MBC, which increased with time (P < 0.001) to 0.36–0.42% at harvest across the treatments (Fig. 6a; Table 1). In contrast, the recovery of added ^15^N in microbial biomass was 26–61% on day two, which decreased (P < 0.001) rapidly to 3–14% with time (Fig. 6b, Table 1). The recovery of ^15^N in dissolved N (DN) also decreased rapidly over time (P < 0.001) (Fig. S2b). Over the study period, fertilization (P < 0.001) and its interaction with tillage (*P* < 0.05), and time (P < 0.01) had a significant impact on ^15^N recovery in microbial biomass (Fig. 6b; Table 1). Fertilization with or without tillage had higher ^15^N recovery in microbial biomass.

### ^13^C and ^15^N recovery in aggregate–size fractions

**Figure 7:**
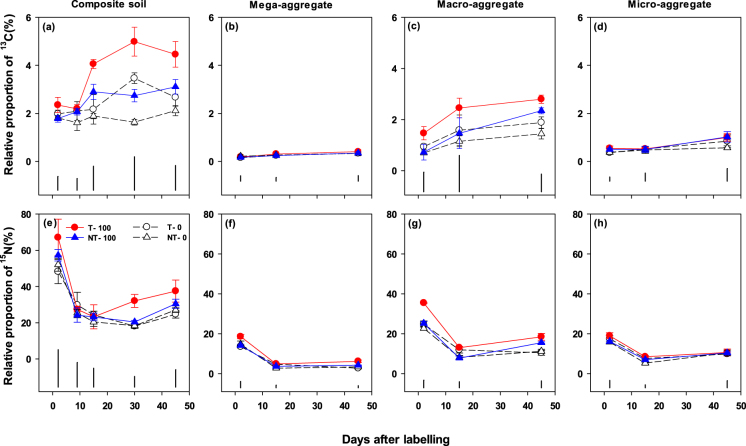
Relative proportion (%) of the pulse-added ^13^CO_2_-C and urea-^15^N recovered in composite soil (**a**,**e**) and in different dry aggregate-size fractions (**b**,**c**,**d** and **f**,**g**,**h**) at 0–0.1 m soil depth from canola flowering to harvesting stage as affected by tillage (T) and no-till (NT) with or without 100 kg urea-N ha^−1^ (*i*.*e*. T-0, T-100, NT-0, NT-100). Error bars are ± standard errors (*n* = 3). Vertical black bars show least significant differences (at 5% level, LSD_0.05_) at different time points.

The ^13^C recovery in whole soil and aggregate-size fractions at 0.1 m depth increased (P < 0.05) over time, while the ^15^N recovery in these soil fractions decreased (P < 0.001) over time (Fig. 7; Table 1). Two days after labelling, the recovery of ^13^C was the highest in macro-aggregates (0.7–1.5%), followed by micro-aggregates (0.4–0.5%) and mega-aggregates (0.15–0.21%). Similarly, the recovery of soil ^15^N in macro-aggregates was the highest (23–35%) on day two, while 16–19% and 14–19% of the ^15^N was recovered in the micro-aggregates and mega-aggregates, respectively (Fig. 7). At harvest, the recovery of ^13^C was in the order of macro-aggregates (1.4–3.0%) > micro-aggregates (0.6–1.0%) > mega-aggregates (0.3–0.45) (Fig. 7). Similarly, the soil ^15^N recovery was 11–18% in macro-aggregates, 10–11% in micro-aggregates, and 3–6% in mega-aggregates. Both tillage (P < 0.05) and N fertilization (P < 0.05) increased the ^13^C recovery in macro-aggregates. However, tillage and fertilization, and their interaction with time had no effects on the ^13^C recovery in mega- and micro-aggregates (Table 1). Both tillage (P < 0.05) and fertilization (P < 0.05), and their interaction with time (P < 0.05) had significant effects on ^15^N recovery in mega- and macro–aggregates, while only tillage (P < 0.05) or fertilization (P < 0.01) had significant effects on ^15^N recovery in micro-aggregates. Overall, T–100 had the highest ^15^N recovery in all the aggregate-size fractions over time.

## Discussion

### Relationships between C and N allocation and assimilation in canola–soil system

This is one of the first field-based studies to provide insights into the impact of tillage intensity and N fertilization on the relationships between belowground C allocation and plant N uptake in a dryland canola crop–soil system. A simultaneous ^13^C– ^15^N isotopic approach was employed to trace the short-term dynamics of new C and N allocation and assimilation at the critical growth stages of canola, *i*.*e*. from flowering to harvest. We found that tillage intensity and N fertilization influenced belowground C allocation, root-microbial interactions and plant N uptake, possibly driven by changes in C source–sink relations among the aboveground and belowground pools^3^. For example, the T–100 resulted in significantly greater new C allocation to the belowground pools and significantly greater new N translocation to the aboveground pools over time, relative to the other treatments (Figs 1, 2 and 3), thereby confirming our first hypothesis. One of the mechanisms for the greatest belowground C allocation and plant N uptake is that the T–100 increased tap root biomass and net ^13^C recovery in both tap and fine roots, relative to any other treatments (Fig. 5), thus suggesting exploration of a greater soil volume, which is likely to enhance N uptake. Root activity and growth can also be associated with simultaneous release of energy rich root exudates, such as carbohydrates, amino acids and organic acids in soil^44, 45^. It is known that these energy rich root exudates are an important source of C for soil microorganisms^46^ stimulating microbial activity and growth, particularly in the root zone, and also in the surface soil layers (Fig. S3) that received a higher proportion of plant C inputs than deeper layers (Fig. 5).

There was a rapid and significant belowground C allocation, as indicated by 4–6% recovery of the ^13^C to 0.3 m depth within two days after pulse labelling (Fig. 3). These results agreed with previous studies that reported immediate allocation of new C to belowground pools, reaching a maximum within one to two days^7, 22, 23^. Our C recovery data in the whole plant–soil system indicate that 21–39% of the added ^13^C was lost from the system within two days (Fig. 3). This could be attributed to a loss of some ^13^CO_2_ during brief opening of the chamber four hours after pulse-labelling, and *via* plant and soil respiration.

Literature suggests that stimulation of microbial activity can facilitate greater microbial uptake of soil-released N^4, 47^. Consistently, our data on the dynamics of microbial ^15^N showed that on day two, the simulated soil-released N was rapidly taken up by microbial biomass (26–61%) across different treatments. Similarly, Grogan and Jonasson^48^ and Nordin *et al*.^49^ reported a significant uptake, 24–47% of soil-applied ^15^N, by soil microbial biomass. We also observed the greatest uptake of ^15^N in microbial biomass in the fertilized *vs*. non-fertilized treatments (*P* < 0.001), which is consistent with our second hypothesis that fertilization will increase soil mineral N uptake by microbes. Overall, tillage (*cf*. NT), and also T–100 (*cf*. NT–100) on day two, enhanced microbial uptake of soil ^15^N in our study, but only at 10% level of significance (Fig. 6; Table 1). As microbial activity will be constrained by N availability in drylands, tillage and tillage–N fertilization may enhance microbial activity and acquisition of SOM-released N for their cell synthesis^50^. After the initial uptake, the decreased recovery of ^15^N in microbial biomass with time (Fig. 6) was most likely due to its rapid turnover and consequent release of microbial-immobilized ^15^N for uptake by plant roots. Greater uptake of soil-released N by plant roots is likely possible when a mutualistic relationship between microbes and roots is enhanced, for example, through implementing management practices that cause greater belowground C allocation, with implications for enhanced N use efficiency, alleviation of N limitation for plant growth, and mitigation of soil N losses^4, 51^. Our study showed significant uptake of soil ^15^N by the plants over time (*i*.*e*. from 5 to 56%), which was greater in the T–100 *cf*. other treatments (Fig. 3). Furthermore, ^15^N retention in the soil at harvest was also greater in the T–100 *cf*. other treatments (Fig. 3). Thus, these results suggest that the T–100 that enhanced microbial activity, including through greater belowground C allocation, possibly facilitated rapid soil N utilisation by microorganisms to meet their N requirements, while enhancing plant N uptake, with potential to minimize N losses *via* leaching and emissions^4, 52^.

In our study, the allocation of new C was the highest in the flower + pod on day two, as also indicated by the ^13^C_atom% excess_ (Fig. S4), which decreased rapidly over time, possibly due to translocation to the other aboveground and belowground pools (Figs 1 and S4), and loss *via* respiration^7, 53, 54^. As belowground C allocation would support plants to take up soil-released N, which is important for the development of aboveground organs, we observed greater translocation of ^15^N to flowers and pods (*cf*. shoots), as indicated by the ^15^N_atom% excess_ across all of the treatments (Fig. S5). Clearly, the T–100 resulted in the highest recovery of soil ^15^N in the seeds at harvest (Fig. 3). Although the fertilized system, whether T or NT, had higher mineral N in the top (Fig. S6b) and deeper soil layers (data not presented), belowground C allocation and plant N uptake were the greatest in T–100 *vs*. NT–100 (Fig. 3). This highlights the importance of tillage (*vs*. soil N availability) in enhancing belowground C allocation, microbial activity, and plant N uptake^43^, while supporting structural and reproductive organs. In this study, we also observed a significant positive correlation (P < 0.05) between seed yield and belowground C allocation, or seed yield and crop N uptake (Fig. 4), and the T–100 resulted in the highest seed yield (Fig. S1). Tillage and N fertilization may also improve canola seed quality through increasing oil and crude protein in seed^14^.

Our study found relatively low belowground C allocation (*i*.*e*. only 4–10% of the ^13^C was allocated belowground to 0.3 m depth at the reproductive stages, *i*.*e*. from flowering to pod filling and maturity; Fig. 3). These results are consistent with other studies that also reported low belowground C allocation at reproductive stages, while allocating a maximum amount of assimilates to aboveground reproductive organs^21, 34, 55, 56^. Additionally, low root-to-shoot C ratio (0.11–0.15) (Table S2) may have caused a net decrease in belowground allocation of ^13^C in our study. In contrast, other studies reported a relatively high allocation of newly assimilated C into belowground pools. For example, depending on plant species, root-to-shoot C ratio (low or high), and plant growth stages (vegetative *vs*. reproductive), up to 10–40% of photo-assimilated C was recovered in soil up to 0.20 m depth^10, 15, 44, 57, 58^.

In the current study, C and N allocation values in the crop–soil were, however, higher than the values reported by another field study in a semi-arid dryland environment, which showed 1.3−1.8% of the new ^13^C allocated belowground to 0.3 m depth and 0.5–0.7% of soil-applied ^15^N translocated aboveground from the flowering to grain harvesting of wheat^7^. The differences could be due to an even distribution of rainfall that resulted in higher gravimetric soil moisture in our study site (8–16% *vs*. 4–8%) than examined by Fang *et al*.^7^ during the C–N chasing period. This might have alleviated the influence of aridity constraints on root activity, belowground C allocation, microbial activity and plant N uptake^7, 18, 59^. Furthermore, canola tends to have a more extensive root system in the top soil layers than wheat in a semi-arid dryland environment^60^.

To our knowledge, there is no field-based study that has examined allocation of newly assimilated C to 1 m soil depth. We found that of the total fine root biomass recovered at harvest, 87–91% was distributed in the top (0–0.3 m) and 9–13% in the deeper (0.3–1 m) soil layers (Fig. 5). Of the total recovered ^13^C in the soil profile in fine roots (*i*.*e*. 9–13% of added ^13^C to 1 m depth), a significant proportion (46%) was recovered in the 0.3–1 m layer (Fig. 3c), despite fine roots biomass in the deeper soil layers was low (Fig. 5). Interestingly, both tap root biomass and ^13^C allocation in tap root and fine root (to 1 m) were higher in the T–100 than the other treatments (Fig. 5). Thus, our study suggests that tillage with N fertilization in the semi-arid region may facilitate more root growth and new belowground C allocation to top and deeper soil layers over time^61^. These plant processes may then assist in better acquisition of nutrients (such as N) and deep soil water to support grain production, while increasing retention of plant-derived C, likely due to lower microbial activity, in deeper soil layers^35, 36^.

### Carbon and nitrogen allocation and stabilization in aggregate-size fractions

Several studies have examined allocation and stabilization of added isotopically-labelled organic C substances in different aggregate-size fractions^26, 62^ that vary in their stability and stabilization of C, for example, micro-aggregates > macro- or mega aggregates^24, 63^. To our knowledge, only Fang *et al*.^7^ reported the impact of management practices on the stabilisation dynamics of belowground ^13^C allocation and soil-released ^15^N in aggregate-size fractions over time under field conditions. Consistent with the study of Fang *et al*.^7^, there were no significant interactive effects of the contrasting tillage and N fertilization practices on the allocation and stabilization of new ^13^C and ^15^N in the aggregate-size fractions (Fig. 7). Meanwhile, we observed rapid (within two days) allocation and distribution of the belowground ^13^C and ^15^N among all aggregate-size fractions, which was higher in macro-aggregates than micro- and mega-aggregates (Fig. 7). This pattern of ^13^C recovery in the aggregate-size fractions could be due greater association of belowground allocated ^13^C, and the fate of most of the fine roots during dry sieving, in macro-aggregates (*vs*. the other aggregate-size fractions), as confirmed by the highest ^13^C_atom% excess_ (Fig. S7). Although ^15^N recovery was highest in macro-aggregates, the relatively high ^15^N_atom% excess_ in mega-aggregates (*cf*. macro- and micro-aggregates) could be due rapid distribution of water-soluble ^15^N in pore spaces of mega-aggregates after soil ^15^N application (Fig. S7). The increased ^13^C recovery among all aggregate-size fractions over time could be related to the pattern of belowground C allocation. Additionally, there could be some retention of new root- and microbial-derived C in all aggregate-size fractions over time^7, 64, 65^. Although the T–100 resulted in higher belowground ^13^C allocation and recovery of the tracer ^15^N in whole soil (Fig. 3) and macro-aggregates (Fig. 7) over time, and also higher total C and N contents in macro-aggregates (Fig. S8), there was no difference in the total C and N stocks in the soil profile to 1 m depth across the management practices at the harvesting stage (Tables S3 and S4). This could be due to rapid decomposition of relatively labile (new inputs) root-derived organic matter by soil microbes with limited stabilization in soil micro-aggregates. Our results showed limited impact of the short-term tillage and N fertilization practices on soil C, soil N and soil aggregate stability (Tables S3 and S4; Fig. S6a). These results agreed with the findings of some other studies that also showed no or limited impact of long-term tillage intensity and/or N fertilization, or other improved management practices (such as reduced tillage and inclusion of pasture in crop rotations) on soil structural stability and/or the accumulation of C and N in soils under semi-arid or subtropical regions in Australia^7, 66, 67^.

In conclusion, our field-based study employed a novel simultaneous stable C and N pulse labelling approach to investigate the influence of contrasting tillage intensity and N fertilization on important coupled plant and soil processes, with implication for N use efficiency and crop yield. We found that during the reproductive stage of canola, management practices that allocated greater C belowground also enhanced plant N uptake. Specifically, tillage along with N fertilization enhanced the activity of belowground pools (*i*.*e*. roots and microbes) to facilitate the linkages between C and N allocation and assimilation, with implication for crop N use efficiency and seed production. In this two-year crop rotation experiment, although macro-aggregates retained more new ^13^C and ^15^N than micro- and mega-aggregates, soil structure stability and SOC stocks were not impacted by tillage intensity and N fertilization. Our research findings suggest that in Australian dryland conditions, tillage (*e*.*g*. when operated to shallow depths) combined with N fertilization can have minimal negative impact on soil structure or other soil properties, while creating a favourable environment for plant and microbial growth/activity, and may enhance belowground C allocation, crop N uptake and seed production of canola. Further research is needed over longer periods, and under different cropping systems, soil types and environments, to determine implications of changes in C–N allocation for soil C accumulation, N use efficiency and crop yield from low-intensity tillage operations.

## Methods

### Site description and experimental design

The field site is located in Wagga Wagga, New South Wales, Australia (35°01′45″S and 147°20′36″E; 210 m a.s.l), with the long-term average rainfall of 541 mm. The soil is a Chromic Luvisol (FAO classification), with a sandy clay loam texture, 5.8 pH, 1.5% total C, and 0.14% total N in the top 0–0.1 m soil layer (see further details in Table S1). Before commencing the tillage–N fertilization experiment in 2012, the site was cropped using no-till or minimal tillage for ~5 years. Canola (*Brassica napus* ‘Hyola 555’) was sown on 20 May 2013 after wheat in the second year of the experiment. The experimental design was a randomised split-plot design with the tillage treatments as the main plot and N application rates as the subplot (5.0 m width × 9.0 m length), replicated three times. Stubble was slashed after the previous crop harvest in both tillage and no-till plots. The tillage plots were cultivated to 0.1 m depth with a scarifier in both directions and then harrowed twice to mix stubble with soil before sowing of canola. At sowing, all plots received 5 kg N ha^−1^ as urea. The remaining amount of urea (95 kg N ha^−1^) was top dressed before stem elongation, 74 days after sowing^14^ (See Supplementary Information, SI).

### *In situ*^15^N and ^13^C pulse labelling

In this study, we selected 12 subplots (5.0 m width × 3.0 m length) across four treatments [0 or 100 kg N ha^−1^ under tillage (T-0 or T-100) or no-till (NT-0 or NT-100)]. Each of the selected subplots was divided into two equal microplots (2.5 m width × 3.0 m length; 10 canola rows each). One microplot was labelled with urea-^15^N at the flowering stage and the other was kept as a control, which did not receive any N or C supplement. For the ^15^N labelling, the soil surface of each microplot was uniformly sprayed with 12.0 l of urea solution at 0.2 g urea-N m^−2^ (99.0 atom% ^15^N, Sercon Ltd, Crewe, UK). Within each of the ^15^N-labelled microplots, a smaller microplot (1.5 m width × 2.0 m length; 6 canola rows) was pulse labelled with ^13^CO_2_ (equivalent to 0.89 g CO_2_-C m^−2^) within 20 h after the application of urea-^15^N.

For the ^13^C pulse labelling, the smaller microplot was sealed with a portable polyvinyl chloride (PVC) chamber (1.5 m width × 2 m length × 1.3 m height), comprising 25 mm thick PVC tube frame covered with 200 µm clear high density polyethylene sheet (Gro-tuff HDPE, 89% light transmission, Cheltenham, Australia). The excess sheet was buried inside soil ditches (10 cm deep) to seal the chamber. The canola plants within each of the sealed chambers were pulse labelled with 5 l ^13^CO_2_ (99.0 atom%, Cambridge Isotope Laboratories, USA). Six microplots across the tillage treatments were labelled on the 9^th^ (^15^N) and 10^th^ (^13^C) September 2013 and the remaining six microplots across the no-till were pulse labelled on the 10^th^ (^15^N) and 11^th^ (^13^C) September. On these two days, the weather was similar (partly cloudy), with the air temperature of 26–27 °C and the minimum and maximum photosynthetic active radiation of *ca*. 400 to 5000 μmol photons m^−2^ s^−1^ during the 4 h ^13^CO_2_ pulse labelling period. Between 11:00 h and 12:00 h, the ^13^CO_2_ was injected at ~500 cm^3^ min^−1^ in each of the chambers through a flow meter (S325-15-170-F/M CO2, Influx Duff and Macintosh, Gascon Systems, Sydney). Chamber air was circulated by a battery-operated mini-fan. The total CO_2_ concentration inside the chamber was monitored using a CO_2_ probe (Vaisala GMP 343, Finland), which dropped to ~250 μl l^−1^ before the injection of ^13^CO_2_ and then temporarily increased to ~500 μl l^−1^ within 10 min after pulse labelling. Between 15:30 and 16:00 h, when the total CO_2_ inside the chamber had dropped to near the compensation point (*ca*. 100–120 μl l^−1^) and the air temperature increased to 38 °C, a portion of the chamber was opened for 30 min to bring the temperature below 30 °C, while increasing the CO_2_ to ~300 μl l^−1^. The chambers were then resealed to capture ^13^CO_2_ respired overnight by canola. The chambers were removed the following morning when the night-accumulated CO_2_ decreased to ~100 μl l^−1^.

### Plant and soil sampling, processing and analysis

Plant and soil samples, representing aboveground and/or belowground pools, were collected before labelling (day zero) and then at 2, 9, 15, 30 and 45 days after labelling. These pools were also collected from the non-labelled plots at seed harvest. At each sampling time, three canola plants were separated into tap roots, leaves, stems, and flowers/pods. At harvest, the pod was separated into pod shell and seed. Seed yield was calculated in t ha^−1^ (see SI Fig. S1). The plant parts were oven-dried at 70 °C soon after sampling, ground using a MM 400 Mixer Mill grinder (Retsch GmbH, Germany) and stored. Biomass yield (g m^−2^) for leaves, stems, flowers/pods (shell and seed at harvest), tap and fine roots was estimated (see SI).

Soil samples were collected on day zero, and then on 2, 9, 15, and 30 days after ^13^C ^15^N labelling using a soil core sampler (cutting head diameter of 6.35 cm), four cores per plot, at 0–0.1, 0.1–0.2, and 0.2–0.3 m depths. At harvest, soil samples were collected from the labelled and control plots to 1 m depth using a hydraulic corer (cutting head diameter of 4.4 cm), six cores per plot, sectioned and composited for each layer (0-0.1, 0.1–0.2, 0.2–0.3, 0.3–0.7 and 0.7–1.0 m). The soil samples were then divided into two portions which were either stored at 4 °C or −18 °C. Fine roots from each soil depth (sampled at day zero, 2, 9, 15, 30 and 45) were separated from the frozen soil by wet sieving and hand picking using tweezers. Soil bulk density was measured at 45 days (Fig. S9).

A subsample of the refrigerated soil was air-dried to *ca*. 20% of water holding capacity within a week, and gently passed through a 6.5 mm sieve. All visible shoot debris, coarse roots and gravels (>2 mm) were removed. Aggregates were separated into five size classes: 2–6.5 mm, 1–2 mm, 0.25–1 mm, 0.25–0.053 mm and <0.053 mm using a Vibratory Sieve-Shaker “Analysette 3”. Mean weight diameter (MWD) of the dry aggregate-size fractions was calculated by multiplying the soil proportion in each aggregate-size class by the mid-point of the size class^68^. Subsamples of macro-aggregates (0.25−2 mm) and micro-aggregates (<0.25 mm) were obtained by mixing the relevant fractions. Subsamples from mega-aggregates ( > 2 mm), macro-aggregates, micro-aggregates and whole soil were further dried at 60 °C for 16 h and ground (<125 μm).

After sieving (<4.75 mm) and removing plant debris, roots and gravels from the refrigerated soil microbial biomass C (MBC) and N (MBN) were measured by the chloroform fumigation-extraction procedure^69, 70^ (see SI). To track the fate of new C and N in microbial biomass, a 20 ml subsample of the 0.125 M K_2_SO_4_ extract (fumigated and non-fumigated) from day 0, 2, 15 and 45 sampling was oven dried at 60 °C and ground. The δ^13^C and δ^15^N of soil microbial biomass were calculated as per An *et al*.^10^ (see SI).

The ground, well-dried, plant (all the aboveground and belowground biomass), soil, (including aggregate) and K_2_SO_4_ samples were analyzed for C%, N%, δ ^13^C and/or δ ^15^N at the University of California’s Stable Isotope Facility, Davis, CA, USA. Standard deviations are 0.02–0.08‰ for δ ^13^C and 0.15–0.30‰ for δ ^15^N using a PDZ Europa 20-20 isotope ratio mass spectrometer (Sercon Ltd., Cheshire, UK). Mineral NH_4_^+^ and NO_3_^−^ were analyzed in the 0.125 M K_2_SO_4_ extracts from the non-fumigated soil samples by a SEAL AQ2 Analyzer (SEAL Analytical, Maquon, WI, USA). The δ ^13^C and δ ^15^N values of the control plant and soil samples (non-labelled; *NL*) collected at different sampling days (*i*.*e*. day zero and 45 days) were similar when comparing across each of the plant or the soil pools (see SI).

### Recovery of added ^13^C and ^15^N in crop–soil system

After calculating the atom% of ^13^C and ^15^N in various measured pools (SI), the following Equations 1–3 were used to estimate ^13^C and ^15^N recovery in the crop–soil system. As calculations are the same for ^13^C and ^15^N, only the ^13^C calculations are presented below.

The enrichment of ^13^C in a sample after pulse labelling at a specific time (^13^C_atom% excess, t_) was calculated:

1 $${}^{13}C_{atom \% \,excess,t}={}^{13}C_{L,t}-{}^{13}C_{NL}$$

where ^13^C_L,t_ is the ^13^C_atom%_ of a sample from the labelled micro-plots at time t (t = two, nine, 15, 30, or 45) and ^13^C_NL_ is the ^13^C_atom%_ of the corresponding sample from the non-labelled (natural abundance) micro-plots.

The amount of ^13^C (g m^−2^) incorporated into each of the aboveground and belowground C pools at time t after pulse labelling (^13^Ci, t) was calculated:

2 $${}^{13}C_{i,t}=\frac{{}^{13}C_{atom \% \,\,excess,t}}{100}\times {C}_{i},t$$

where C_*i*,*t*_ is the amount of C (g m^−2^) in each of the measured pools (*i* = leaf, stem, pods, pod shell, seed, tap root, fine root, soil, soil microbial biomass) at time *t*.

The weighted ^13^C recovery (^13^C*rec*_*i*, *t*_ %) in each of the measured C pools at time *t* after pulse labelling was calculated as the percentage of total mass of added ^13^C (g C m^−2^) *via* pulse labelling.

3 $${}^{13}Cre{c}_{i,t}=\frac{{}^{13}C_{i,t}}{{}^{13}C_{added}}\times 100$$

The total ^13^C recovery (%) in the crop–soil system at a specific time was calculated by summing ^13^Crec_*i*,*t*_ across all pools and/or soil depths except for the ^13^Crec_*i*,*t*_ in the microbial biomass C (MBC) and dissolved organic C (DOC) pools, which is part of the soil ^13^C.

The calculations for the amount (g N m^−2^) of C and N in plant biomass, microbial biomass, and soil pools, including in aggregate-size fractions, are presented in SI.

### Statistical analysis

Repeated measures analysis was performed for ^13^C and ^15^N partitioning in aboveground and belowground pools and soil properties over time using a linear mixed model. Each analysis consisted of fixed effects of tillage, fertilizer, time, and all interactions, and random effects of replicate, main plot (defined as tillage by replicate) and their interactions with time, and either plot effects or autoregressively correlated residual errors within plots, and with heterogeneous residual variances varying with time. For C and N stocks in pools, a linear mixed model was fitted with fixed effects of tillage, fertilizer and their interactions, and random effects of replicate and main plot. The degree of relationships between seed yield and belowground C allocation, or crop N uptake (*i*.*e*. aboveground N allocation), was examined using scatter plots with associated (Pearson) correlations. All models were fitted in the ASReml statistical package^71^ within the R statistical software environment^72^. Wald-type F statistics were calculated for all fixed terms (tillage, fertilizer, time and all associated interactions); predicted means for fertilizer by tillage by time are shown with 5% LSD at each time point.

## Electronic supplementary material

Supplementary Information
