# Supplementary material for: Tillage and nitrogen fertilization enhanced belowground carbon allocation and plant nitrogen uptake in a semi-arid canola crop–soil system
Source: Sci Rep. 2017 Sep 6;7:10726. doi: 10.1038/s41598-017-11190-4 (PMC5587530; doi:10.1038/s41598-017-11190-4)
Supplement: Supplementary file 1 — Supplementary Information [file 41598_2017_11190_MOESM1_ESM.doc]

**Supplementary Information**

**Tillage and nitrogen fertilization enhanced belowground carbon allocation and plant nitrogen uptake in a semi-arid canola crop–soil system**

Jharna Rani Sarker^1,2^*, Bhupinder Pal Singh^1,2^,*, Xinhua He^3^, Yunying Fang^2^, Guangdi D Li^4^, Damian Collins^2^, Annette L Cowie^1,5^

^1^ University of New England, Armidale, NSW 2351, Australia; ^2^ NSW Department of Primary Industries, Elizabeth Macarthur Agricultural Institute, Woodbridge Rd, Menangle, NSW 2568, Australia; ^3^ College of Resources and Environment, Southwest University, Chongqing 400715, China; ^4^ NSW Department of Primary Industries, Wagga Wagga Agricultural Institute, Wagga Wagga, NSW 2650, Australia; ^5^ NSW Department of Primary Industries, Beef Industry Centre, Trevenna Road, Armidale, NSW 2351, Australia

Author for correspondence:

Tel.: [+61 2 4640 6406](tel:%2B61 2 4640 6451)

E-mail address: [bp.singh@dpi.nsw.gov.au](mailto:bp.singh@dpi.nsw.gov.au) (B.P. Singh)

* These authors contributed equally to this work.

**Methods**

**Site description and experimental design**. Long-term average rainfall at this site is 541 mm and is relatively evenly distributed throughout the year. In summer, daily evaporation is 6–8 mm, which is higher than the rainfall. Canola (*Brassica* *napus* 'Hyola 555') was grown in the second year (sown on 20 May 2013) at 4 kg/ha using an air-seeder with knife points fitted to the front of the tynes, spaced 0.25 m apart. The tillage (T) plots were cultivated to 0.1 m depth with a scarifier in both directions and then harrowed twice before sowing, no cultivation for the no-till plots prior to sowing. At sowing all plots received 5 kg N ha^-1^ as urea and 15 kg P ha^-1^ as superphosphate (8.8% P and 11% sulphur) as base fertilisers (see further details in Li *et al.*^1^.

***In situ* ^15^N and ^13^C labelling.** For ^13^C labelling, each portable chamber was covered with 200 µm clear high density polyethylene sheet and excess of 0.5 m sheet along all the four sides of the chamber was buried inside soil ditches (0.1 m deep) and covered with moist soil to ensure complete sealing. In each chamber the ^13^CO_2_ injection port was aligned next to one of the two battery-operated mini-fans to ensure uniform circulation of ^13^CO_2_ and air inside the sealed chamber.

**Plant and soil sampling, processing and analysis.** The plant biomass was estimated as g per m^2^ by measuring the total dry (70°C) biomass from two rows within 2 m outside the experimental plots on 0, 2, 9, 15, and 30 days, and then inside the labelled plots (the middle four canola rows) at harvest.

On day 0, 2, 9, 15, and 30 after ^13^C^15^N labelling, two soil samples were collected from on-rows (next to the plant stem) and two from inter-rows and then composited for 0–0.1, 0.1–0.2 and 0.2–0.3 cm depth separately. At harvest, six intact soil cores (three each from on- and inter-rows) from 0-1.0 m depth were collected using a hydraulic corer (with a cutting head diameter of 4.4 cm) and the soil cores were sectioned and composited layer-wise (0-0.1, 0.1–0.2, 0.2–0.3, 0.3–0.7 and 0.7–1.0 m).

The δ^13^C and δ^15^N values of the control plant and soil samples (non-labelled; *NL*) collected at different sampling days (*i.e.* at day zero and 45) were similar (see below) when comparing across each of the plant or the soil pools. The δ^13^C values (across the replicated plots) of the control aboveground pools at day zero were in the range of -31.2 to -31.6‰ in leaf, -30.4 to -30.7‰ in stem and -30.4 to -30.8‰ in flower + pod. At harvest the δ^13^C values of the control aboveground pools were in the range of -30.9 to -31.3‰ in leaf, -29.8 to -30.4‰ in stem, -30.0 to -30.3 ‰ in pod shell and -30.2 to -30.5‰ in seed. The δ^15^N values of the control aboveground pools at day zero were in the range of 6.0 to 7.2‰ in leaf, 4.8 to 6.4‰ in stem and 5.2 to 7.2‰ in flower + pod. At harvest the δ^15^N values of the control aboveground pools were in the range of 6.4 to 7.6‰ in leaf, 5.1 to 7.0‰ in stem, 5.8 to 7.8 ‰ in pod shell and 6.0–7.7‰ in seed. The δ^13^C values of the control belowground pools at day zero were in the range of -29.8 to -30.3‰ in tap root, -27.4 to -28.6‰ in fine roots to 0–0.3 m depth and -25.9 to -26.2‰ in soils to 0–0.3 m depth. At harvest the δ^13^C values of the control belowground pools were in the range of -29.5 to -29.9‰ in tap root, -27.1 to -28.0‰ in fine roots to 0–0.3 m depth and -25.3 to -25.8‰ in soils to 0–1m depth. The δ^15^N values of the control belowground pools at day zero were in the range of 1.7 to 3.7‰ in tap root, 1.8 to 3.8‰ in fine roots to 0–0.3 m depth and 5.4 to 6.6‰ in soils to 0–0.3 m depth. At harvest the δ^15^N values of the control belowground pools were in the range of 2.1 to 3.3‰ in tap root, 2.3 to 4.4‰ in fine roots to 0–0.3 m depth and 5.0 to 7.2‰ in soils to 0–1 m depth.

**Chloroform fumigation-extraction procedure.** The fumigated soil (~20 g) and an equivalent amount of non-fumigated soil on the day of fumigation were extracted with 80 ml of 0.125 M K_2_SO_4_ for 1 h, and supernatant was filtered through a glass-fibre filter (Whatmann GF/C) and stored at –18°C. Total C and N in the fumigated and non-fumigated extracts were analysed using a Shimadzu Analyser (TOC-L CPH/CPN, Japan). Microbial biomass C and N were calculated as the difference between extractable C and N from the fumigated and non-fumigated soils, respectively. The conversion factor of 0.45 for biomass C^1^ and 0.54 for biomass N were then applied^3^.

**Soil microbial biomass ^13^C or ^15^N.** To determine the
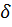
 ^13^C_MBC_ (‰) in each sample the following mass balance equation was used^4^:


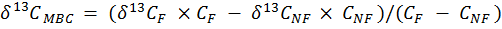
 (1)

Where
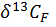
(‰) and
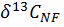
(‰) are the
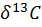
 values of the fumigated and non-fumigated K_2_SO_4_ extracts, respectively and
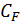
and
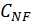
refer to the mass of dissolved organic C (g C m^-2^) from the fumigated and non-fumigated K_2_SO_4_ extracts, respectively. The same *Eq.* 1 was used for
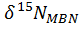
.

A**tom% ^13^C or ^15^N in crop and soil pools.** The isotopic ratio ^13^C:^12^C or ^15^N:^14^N of each sample (R_sample_) was calculated:


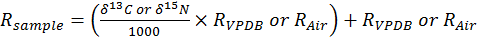
 (2)

where, *R_VPDB_* is the ratio of ^13^C:^12^C (0.0111796) in Vienna Pee Dee Belemnite and *R_Air_* is the ratio of ^15^N:^14^N (0.0036765) in atmospheric N.

The ^13^C_atom%_ or ^15^N_atom%_ (% of total C or N atoms) of each sample was determined as below.


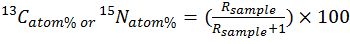
 (3)

**Plant biomass C or N pool (g m^-2^).** The plant biomass C or N pool was calculated as the mass of biomass (g m^-2^) multiplied by biomass C or N content (%), respectively.

**Microbial biomass C or N pool (g m^-2^).** The following equation was used to calculate the soil MBC pool.


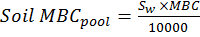
 (4)

Where
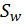
is the mass of soil (kg m^-2^) and MBC is the microbial biomass C (mg kg^-1^ soil). The same *Eq.* 4 was used for
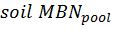


**Soil C pool (g m^-2^).** The total soil C pool in each of the soil layers across tillage and fertilization treatments was calculated as below.


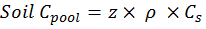
 (5)

where z (m) is the thickness of the considered soil layer,
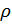
 (g m^-3^) is the bulk density, and
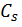
is the total C content (%) in whole soil. The same *Eq.* 5 was used for
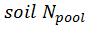
.

**Soil aggregate C pool (g m^-2^).** The total C pool in each soil aggregate-size fraction at 0–10 cm depth was calculated as below.


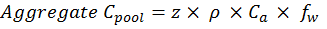
 (6)

Where
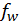
 and
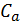
 are the proportion (in whole soil) and total C content (%) of a specific aggregate-size fraction. The same *Eq.* 6 was used in
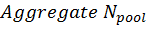
.

Both total soil C and N pools to 1 m depth were expressed at the harvest time as stocks in t ha^-1^ on a minimum equivalent soil mass basis^5^ across the treatments after subtraction of total C and N stocks (t ha^-1^) in fine roots.

**Table S1. Baseline soil chemical and physical properties at the experimental plots from different depths.**

| Soil depths | 0–10 cm | 10–20 cm | 20–30 cm |
| --- | --- | --- | --- |
| Total carbon (%) | 1.55±0.09 | 0.68±0.06 | 0.51±0.06 |
| Total nitrogen (%) | 0.14±0.01 | 0.07±0.00 | 0.06±0.00 |
| Carbon-to-nitrogen | 11.1±0.2 | 9.9±0.4 | 8.7±0.5 |
| pH_1:5 water_ | 5.8±0.03 | 5.6±0.1 | 6.5±0.1 |
| EC_1:5 water_ (µS/cm) | 147.1 ±9.7 | 123.0 ±3.9 | 95.0 ±1.6 |
| Bulk density | 1.2±0.02 | 1.4±0.02 | 1.5±0.01 |
| Sand (%) | 61.2±0.6 | 52.1±1.8 | 49.1±2.0 |
| Silt (%) | 12.5±0.6 | 9.4±1.8 | 8.1±1.2 |
| Clay (%) | 26.3±0.7 | 38.4±0.1 | 42.7±1.6 |
| Textural class | Sandy clay loam | Sandy clay | Sandy clay |

EC = Electrical conductivity. The numbers after ‘**±**’ are the standard errors (n = 3).

**Table S2. Grain-to-shoot and root-to-shoot C ratios under tillage (T) and no-till (NT) with or without 100 kg urea-N ha^-1^ (*i.e.*** T-0, T-100, NT-0, NT-100) at the harvesting stage of canola.

| Treatments | Ratio of C mass | |
| --- | --- | --- |
|  | Grain-to-shoot | Root-to-shoot |
| T–0 | 0.21 ± 0.03 | 0.11 ± 0.03 |
| T–100 | 0.28 ± 0.04 | 0.13 ± 0.01 |
| NT–0 | 0.25 ± 0.02 | 0.15 ± 0.01 |
| NT–100 | 0.24 ± 0.03 | 0.14 ± 0.00 |

The numbers after ‘**±**’ are the standard errors (n = 3).

**Table S3.** **Total carbon (C) stocks in aboveground and belowground pools, with the soil C stocks expressed on a minimum equivalent soil mass basis, after subtraction of C stocks in roots up to 1 m depth, under tillage (T) and no-till (NT) with or without 100 kg urea-N ha^-1^ (*i.e.* T-0, T-100, NT-0, NT-100) at the harvesting stage of canola.**

|  | Depth (m) | T-0 | T-100 | NT-0 | NT-100 |
| --- | --- | --- | --- | --- | --- |
|  |  | C stocks (t ha^-1^) | | | |
| Crop residue |  | 2.7 ± 0.2 | 3.4 ± 0.5 | 2.6± 0.1 | 2.8 ± 0.2 |
| Grain |  | 0.6 ± 0.03 | 0.9 ± 0.05 | 0.6 ± 0.04 | 0.7 ± 0.06 |
| Tap root |  | 0.18 ± 0.04 | 0.34 ± 0.04 | 0.26 ± 0.01 | 0.28 ± 0.02 |
| Fine roots | 0–0.1 | 0.13 ± 0.02 | 0.14 ± 0.01 | 0.13 ± 0.01 | 0.15 ± 0.00 |
|  | 0.1–0.2 | 0.04 ± 0.00 | 0.04 ± 0.00 | 0.04 ± 0.00 | 0.04 ± 0.00 |
|  | 0.2–0.3 | 0.03 ± 0.00 | 0.04 ± 0.00 | 0.04 ± 0.00 | 0.03 ± 0.00 |
|  | 0.3–0.7 | 0.02 ± 0.00 | 0.02 ± 0.00 | 0.03 ± 0.00 | 0.02 ± 0.00 |
|  | 0.7–1 | 0.009 ± 0.00 | 0.004 ± 0.00 | 0.007 ± 0.00 | 0.004 ± 0.00 |
| Soil without roots | 0–0.1 | 18.5±0.8 | 20.2±2.7 | 16.8±0.0 | 16.0±0.5 |
|  | 0.1–0.2 | 8.9±0.6 | 9.2±1.6 | 7.6±0.1 | 7.3±0.3 |
|  | 0.2–0.3 | 7.1±0.4 | 9.0±1.3 | 5.6±0.3 | 5.4±0.2 |
|  | 0.3–0.7 | 10.0±0.6 | 9.8±1.4 | 9.5±1.0 | 8.6±0.4 |
|  | 0.7–1 | 3.2±0.7 | 3.1±0.3 | 2.2±0.3 | 2.6±0.1 |

The numbers after ‘**±**’ are the standard errors (n = 3).

**Table S4.** **Total nitrogen (N) stocks in aboveground and belowground pools, with the soil N stocks expressed on a minimum equivalent soil mass basis, after subtraction of N stocks in roots up to 1 m depth, under tillage (T) and no-till (NT) with or without 100 kg urea-N ha^-1^ (*i.e.* T-0, T-100, NT-0, NT-100) at the harvesting stage of canola.**

|  | Depth (m) | T-0 | T-100 | NT-0 | NT-100 |
| --- | --- | --- | --- | --- | --- |
|  |  | N stocks (t ha^-1^) | | | |
| Crop residue |  | 0.05 (0.01) | 0.08 ± 0.01 | 0.07 ± 0.09 | 0.09 ± 0.01 |
| Grain |  | 0.03 ± 0.00 | 0.06 ± 0.01 | 0.04 ± 0.00 | 0.05 ± 0.01 |
| Tap root |  | 0.003 ± 0.00 | 0.002 ± 0.00 | 0.004 ± 0.00 | 0.005 ± 0.00 |
| Fine roots | 0–0.1 | 0.0072 ± 0.00 | 0.0077 ± 0.00 | 0.0065 ± 0.00 | 0.0079 ± 0.00 |
|  | 0.1–0.2 | 0.0014 ± 0.00 | 0.0018 ± 0.00 | 0.0018 ± 0.00 | 0.0016 ± 0.00 |
|  | 0.2–0.3 | 0.0015 ± 0.00 | 0.0016 ± 0.00 | 0.0015 ± 0.00 | 0.0012 ± 0.00 |
|  | 0.3–0.7 | 0.0008 ± 0.00 | 0.0009 ± 0.00 | 0.0009 ± 0.00 | 0.0008 ± 0.00 |
|  | 0.7–1 | 0.0004 ± 0.00 | 0.0002 ± 0.00 | 0.0002 ± 0.00 | 0.0002 ± 0.00 |
| Soil without roots | 0–0.1 | 1.58 ± 0.13 | 1.84 ± 0.26 | 1.71 ± 0.02 | 1.66 ± 0.05 |
|  | 0.1–0.2 | 0.85 ± 0.05 | 0.91 ± 0.12 | 0.84 ± 0.02 | 0.84 ± 0.03 |
|  | 0.2–0.3 | 0.77 ± 0.04 | 0.91 ± 0.05 | 0.75 ± 0.02 | 0.73 ± 0.01 |
|  | 0.3–0.7 | 1.34 ± 0.01 | 1.32 ± 0.09 | 1.25 ± 0.07 | 1.19 ± 0.01 |
|  | 0.7–1 | 0.56 ± 0.03 | 0.53 ± 0.02 | 0.46 ± 0.02 | 0.51 ± 0.00 |

The numbers after ‘**±**’ are the standard errors (n = 3).

**Table S5. Results of repeated-measures ANOVA (P values) to test for overall effects of tillage, fertilizer, time and their interaction on ^13^C and ^15^N atom% excess in aboveground biomass.**

|  | Tillage | Fertilizer | Time | Tillage × Fertilizer | Tillage × Time | Fertilizer × Time | Tillage × Fertilizer × Time |
| --- | --- | --- | --- | --- | --- | --- | --- |
| **^13^C atom% excess** |  |  |  |  |  |  |  |
| Leaf | 0.504 | 0.359 | **0.013** | 0.853 | 0.078 | 0.238 | **0.003** |
| Stem | 0.663 | 0.266 | **0.024** | 0.587 | 0.902 | 0.381 | 0.803 |
| Flower + pod | 0.673 | **0.006** | **<0.001** | 0.809 | 0.267 | **0.004** | 0.901 |
| Pod shell + seed(day 45) | 0.192 | **0.017** | **-** | 0.952 | - | - | - |
|  |  |  |  |  |  |  |  |
| **^15^N atom% excess** |  |  |  |  |  |  |  |
| Leaf | **0.018** | **0.006** | **<0.001** | 0.477 | 0.594 | 0.481 | 0.414 |
| Stem | **<0.001** | **0.006** | **<0.001** | **<0.001** | 0.115 | 0.083 | **0.004** |
| Flower + pod | **<0.001** | **<0.001** | **<0.001** | **0.001** | **0.006** | **0.031** | **0.028** |
| Pod shell + seed(day 45) | **0.026** | 0.975 | - | 0.092 | - | - | - |

Bold values highlight significant effects at P<0.05.

Table S6. Results of repeated-measures ANOVA (P values) to test for overall effects of tillage, fertilizer, time and their interactions on ^13^C and ^15^N atom% excess and total C and N contents in composite soil and aggregate-sizes fractions and soil physical, chemical and biological properties.

|  | Tillage | Fertilizer | Time | Tillage × Fertilizer | Tillage × Time | Fertilizer × Time | Tillage × Fertilizer × Time |
| --- | --- | --- | --- | --- | --- | --- | --- |
| **^13^C atom% excess** |  |  |  |  |  |  |  |
| Composite soil (0−10 cm) | 0.539 | 0.411 | **0.006** | 0.675 | 0.544 | **0.007** | 0.290 |
| Mega-aggregate (> 2 mm) | 0.992 | 0.703 | **0.017** | 0.124 | 0.865 | 0.924 | 0.898 |
| Macro-aggregate (0.25–2 mm) | 0.183 | 0.063 | **0.001** | 0.544 | 0.185 | 0.289 | 0.176 |
| Micro-aggregate (<0.25 mm) | 0.361 | 0.607 | **0.003** | 0.889 | 0.687 | 0.619 | 0.502 |
|  |  |  |  |  |  |  |  |
| **^15^N atom% excess** |  |  |  |  |  |  |  |
| Composite soil (0−10 cm) | 0.337 | **<0.001** | **<0.001** | **0.001** | 0.727 | 0.075 | 0.097 |
| Mega-aggregate (> 2 mm) | **0.001** | **0.024** | **<0.001** | **0.003** | **<0.001** | **0.044** | **0.002** |
| Macro-aggregate (0.25–2 mm) | **<0.001** | **0.013** | **<0.001** | 0.084 | **0.006** | **0.035** | 0.863 |
| Micro-aggregate (<0.25 mm) | **0.034** | 0.077 | **<0.001** | 0.846 | 0.118 | 0.212 | 0.196 |
|  |  |  |  |  |  |  |  |
| **Soil total C content (%)** |  |  |  |  |  |  |  |
| Composite soil (0−10 cm) | 0.324 | **0.022** | **<0.001** | **0.005** | **<0.001** | 0.440 | 0.190 |
| Mega-aggregate (> 2 mm) | 0.317 | **0.014** | 0.065 | 0.970 | 0.363 | 0.393 | **0.005** |
| Macro-aggregate (0.25–2 mm) | **0.006** | **0.024** | 0.061 | 0.314 | 0.868 | 0.489 | 0.593 |
| Micro-aggregate (<0.25 mm) | **0.008** | **0.005** | **0.047** | 0.548 | 0.220 | **0.025** | 0.824 |
|  |  |  |  |  |  |  |  |
| **Soil total N content (%)** |  |  |  |  |  |  |  |
| Composite soil (0−10 cm) | 0.165 | **0.032** | **0.004** | **0.019** | **0.020** | 0.692 | 0.159 |
| Mega-aggregate (> 2 mm) | 0.461 | 0.382 | 0.865 | **<0.001** | 0.690 | 0.485 | 0.728 |
| Macro-aggregate (0.25–2 mm) | 0.463 | **0.012** | 0.144 | **0.005** | 0.834 | 0.170 | 0.426 |
| Micro-aggregate (<0.25 mm) | 0.143 | **0.016** | **0.019** | 0.171 | 0.393 | 0.881 | 0.364 |

Bold values highlight significant effects at P<0.05

**Table S7. Results of repeated-measures ANOVA (P values) to test for overall effects of tillage, fertilizer, time and their interactions on soil physical, chemical and biological properties.**

|  | Tillage | Fertilizer | Time | Tillage × Fertilizer | Tillage × Time | Fertilizer × Time | Tillage × Fertilizer × Time |
| --- | --- | --- | --- | --- | --- | --- | --- |
| DMWD (0–10 cm) | 0.261 | 0.740 | **<0.001** | 0.430 | **0.050** | 0.091 | 0.552 |
| DOC (0–10 cm) (g/m2) | 0.151 | **0.024** | **0.001** | 0.707 | 0.448 | 0.501 | 0.385 |
| DN (0–10 cm) (g/m2) | 0.531 | **0.037** | **0.027** | 0.085 | 0.591 | **<0.001** | 0.377 |
| MBC (0–10 cm) (g/m2) | 0.067 | **<0.001** | **0.047** | 0.534 | 0.401 | 0.972 | 0.694 |
| MBN (0–10 cm) (g/m2) | 0.062 | **<0.001** | **<0.001** | 0.874 | 0.689 | **0.021** | 0.782 |
| Mineral N (0–10 cm) (g/m2) | 0.425 | **<0.001** | **0.009** | 0.824 | 0.449 | 0.162 | 0.462 |

Bold values highlight significant effects at P<0.05. DMWN = Dry mean weight diameter.

**Figure captions**

**Figure S1. Biomass yield (t ha^-1^) from canola flowering to harvesting (a) and seed yield (t ha^-1^) of canola at harvesting stage (b) as affected by tillage (T) and no-till (NT) with or without 100 kg urea-N ha^-1^ (*i.e.* T-0, T-100, NT-0, NT-100).** Error bars are ± standard errors (*n* = 3). Vertical black bar show least significant differences (at 5% level, LSD_0.05_) at harvest.

**Figure S2. Relative proportion (%) of the pulse-added ^13^CO_2_-C in dissolved organic carbon (DOC) (a), urea-^15^N in dissolved nitrogen (DN) (b), and dissolved organic carbon (g m^-2^) (c), dissolved nitrogen (g m^-2^) (d) in the 0–0.1 m soil from canola flowering to harvesting stage as affected by tillage (T) and no-till (NT) with or without 100 kg urea-N ha^-1^ (*i.e.* T-0, T-100, NT-0, NT-100).** Error bars are ± standard errors (*n* = 3). Vertical black bars show least significant differences (at 5% level, LSD_0.05_) at different time points.

**Figure S3. Soil microbial biomass carbon (MBC) (g m^-2^) and microbial biomass nitrogen (MBN) (g m^-2^) at 0−0.1 m depth (a, d), 0.1−0.2 m depth (b, e) and 0.2−0.3 m depth (c, f) from canola flowering to harvesting stage as affected by tillage (T) and no-till (NT) with or without 100 kg urea-N ha^-1^ (*i.e.* T-0, T-100, NT-0, NT-100).** Error bars are ± standard errors (*n* = 3). Vertical black bars show least significant differences (at 5% level, LSD_0.05_) at different time points.

**Figure S4. ^13^C_atom%_ excess in the aboveground (a, b, c) and belowground pools (d, e, f) ) in a canola crop–soil system from flowering to harvesting as affected by tillage (T) and no-till (NT) with or without 100 kg urea-N ha^-1^ (*i.e.* T-0, T-100, NT-0, NT-100).** Error bars are ± standard errors (*n* = 3). Vertical black bars show least significant differences (at 5% level, LSD_0.05_) at different time points.

**Figure S5.** ^1^**^5^N_atom%_ excess in the aboveground (a, b, c) and belowground pools (d, e, f) in a canola crop–soil system from flowering to harvesting as affected by tillage (T) and no-till (NT) with or without 100 kg urea-N ha^-1^ (*i.e.* T-0, T-100, NT-0, NT-100).** Error bars are ± standard errors (*n* = 3). Vertical black bars show least significant differences (at 5% level, LSD_0.05_) at different time points.

**Figure S6. Mean weight diameter (mm) of dry soil aggregates (a) and soil mineral N (g m^-2^) (b) at 0−0.1 m depth soil from canola flowering to harvesting as affected by tillage (T) and no-till (NT) with or without 100 kg urea-N ha^-1^ (*i.e.* T-0, T-100, NT-0, NT-100).** Error bars are ± standard errors (*n* = 3). Vertical black bars show least significant differences (at 5% level, LSD_0.05_) at different time points.

**Figure S7. ^13^C_atom%_ and ^15^N _atom%_ excess in composite soil (a, e) and different dry aggregate-size fractions (b, c, d and f, g, h) at 0–0.1 m soil depth from canola flowering to harvesting stage as affected by tillage (T) and no-till (NT) with or without 100 kg urea-N ha^-1^ (*i.e.* T-0, T-100, NT-0, NT-100).** Error bars are ± standard errors (*n* = 3). Vertical black bars show least significant differences (at 5% level, LSD_0.05_) at different time points.

**Figure S8. Total C and N content (%) in composite soil (a, e) and different dry aggregate-size fractions (b, c, d and f, g, h) at 0–0.1 m soil depth from canola flowering to harvesting stage as affected by tillage (T) and no-till (NT) with or without 100 kg urea-N ha^-1^ (*i.e.* T-0, T-100, NT-0, NT-100).** Error bars are ± standard errors (*n* = 3). Vertical black bars show least significant differences (at 5% level, LSD_0.05_) at different time points.

**Figure S9. Bulk density of soil at different depths (0–0.1, 0.1–0.2, 0.2–0.3, 0.3–0.7 and 0.7–1 m) at harvesting stage under different management practices as affected by tillage (T) and no-till (NT) with or without 100 kg urea-N ha^-1^ (*i.e.* T-0, T-100, NT-0, NT-100).** Error bars are ± standard errors (*n* = 3). Vertical black bars show least significant differences (at 5% level, LSD_0.05_) at different depths.

**Figure S10. Total C and N mass (g m^-2^) in composite soil (a, e) and different dry aggregate-size fractions (b, c, d and f, g, h) at 0–0.1 m soil depth from canola flowering to harvesting stage as affected by tillage (T) and no-till (NT) with or without 100 kg urea-N ha^-1^ (*i.e.* T-0, T-100, NT-0, NT-100).** Error bars are ± standard errors (*n* = 3). Vertical black bars show least significant differences (at 5% level, LSD_0.05_) at different time points.

**Figure S1.**

**Figure S2.**

**Figure S3.**

**Figure S4.**

**Figure S5.**

**Figure S6.**

**Figure S7.**

**Figure S8**

**Figure S9**

**Figure S10.**

**References**

1. Li, G. D. *et al*. Tillage does not increase nitrous oxide emissions under dryland canola (*Brassica napus* L.) in a semiarid environment of south-eastern Australia. *Soil Res.* **54**, 512–522 (2016).
2. Wu, J., Joergensen, R., Pommerening, B., Chaussod, R. & Brookes, P. Measurement of soil microbial biomass C by fumigation-extraction—an automated procedure. *Soil Biol. Biochem.* **22**, 1167–1169 (1990).
3. Brookes, P. C., Landman, A., Pruden, G. & Jenkinson, D. S. Chloroform fumigation and the release of soil nitrogen: a rapid direct extraction method to measure microbial biomass nitrogen in soil. *Soil Biol. Biochem.***17**, 837-842 (1985).
4. An, T. T. *et al*. Carbon fluxes from plants to soil and dynamics of microbial immobilization under plastic film mulching and fertilizer application using ^13^C pulse-labeling. *Soil Biol. Biochem.* **80**, 53–61 (2015).
5. Lee, J., Hopmans, J. W., Rolston, D. E., Baer, S. G. & Six, J. Determining soil carbon stock changes: simple bulk density corrections fail. *Agric. Ecosys. Environ.* **134**, 251–256 (2009).
